# Biochemical Serum Markers Influencing Maternal Age Risk for Down's Syndrome in Quadruple Marker

**DOI:** 10.7759/cureus.23555

**Published:** 2022-03-27

**Authors:** Zehratul Quresh., Chinni Dharavath

**Affiliations:** 1 Clinical Biochemistry, Apollo Diagnostics, Hyderabad, IND

**Keywords:** down's syndrome, amniocentesis, quadruple marker, second trimester screen, aneuploidy, maternal age, trisomy 21

## Abstract

Objective: Maternal age is the primary risk factor associated with Down syndrome (DS) in the fetus. Biochemical serum markers in maternal screenings have improved DS detection rates in prenatal screenings. However, there is a dilemma regarding which age group should undergo preliminary noninvasive screening before undergoing invasive diagnostic procedures. Based on recommendations, all pregnancies are at risk of chromosomal abnormalities. While all women should be offered screenings, those over 35 are mainly offered an invasive diagnostic procedure, and serum screening tests are of little benefit for this age group. This study evaluated the maternal serum screening population and the significance of the final screen positivity rate in the risk group aged above 35 years.

Method: An observational retrospective study was conducted on a cohort of pregnancies in the second trimester (14-20 weeks and 6 days of gestation) over a period of one year. The quadruple test consisted of serum alpha-fetoprotein (AFP), free beta hCG, unconjugated estriol e3 (Ue3), and inhibin-A. The risk for DS was calculated using software with corrections for ethnicity, smoking, weight, and age. We compared the age risk for DS with the biochemical risk. Statistical analysis was done using McNemar’s test to test the proportion of screen-positive (SP) cases between the two calculation methods, i.e., age alone versus final risk calculation with biomarkers.

Results: The proportion of SP cases from age risk and final risk were 56.3% and 12.6%, respectively. The computed McNemar’s chi-square test statistic was 97.959 (p < 0.001), which showed a significant reduction in SP cases when biomarkers were added to screen for trisomy 21 women aged >35 years.

Conclusion: The age risk of DS increased with increasing maternal age. Notably, the final biochemical risk in this population was significantly lower. Consequently, we proposed that a noninvasive serum screening be used to screen all age groups to rule out negative screen cases before subjecting them to invasive procedures.

## Introduction

Down's syndrome (DS), or trisomy 21 (T21), is an additional copy of chromosome 47 instead of the standard 46 in all cells or a few cells, leading to the variability of expression severity in the fetus. DS is a leading cause of intellectual disability, which can be prevented via screening programs and diagnostic methods to identify pregnancies at risk, thereby providing an opportunity for the parents to make an informed decision.

The incidence of DS, which is 1 in 800 [1] and 1 in 700 live births, has been reported to be directly affiliated with maternal age for decades [2]. Studies in England and Wales have shown that with the increasing availability of noninvasive serum screening tests, a higher proportion of DS fetuses are detected prenatally, especially among younger mothers (the proportion of mothers aged <35 years who were diagnosed prenatally with DS increased from 10% to 60% in England and Wales from 1989 to 2010) [3].

Maternal screening for DS dates back to the 1970s. In the 1980s, biochemical parameters and ultrasound markers were added to this screening to estimate the risk of DS, which not only improved the detection rate but also helped in understanding DS risk in the lower age group [4]. However, advanced pregnancies were still offered an invasive diagnostic method over screening.

Screening and detection modalities of DS in the second trimester

Noninvasive Maternal Screening Modalities

The hallmark of a screening test is to rule out certain diseases and avoid expensive, invasive procedures. However, this depends greatly on the sensitivity of the screening tests. Multiple screening strategies are recommended to rule out DS prenatally. One of the most common approaches for assessing the risk of DS is second-trimester screening [5].

A quadruple test in the second trimester that uses four serum markers, namely, (i) alpha-fetoprotein (AFP), (ii) human chorionic gonadotropin (beta hCG), (iii) unconjugated estriol (Ue3), and (iv) inhibin-A, has a detection rate of 81-83% with a false-positive rate of 5% according to a prospective US-based study, FASTER [6,7] and the UK-based SURUSS trials [7].

Generally, the first-trimester screening test is increasingly preferred. Patients undergo first-trimester combined screening, and screen positives at this stage are offered chorionic villus sampling. Further, patients with negative test results return after 14 weeks for quadruple marker tests. At this stage, both the first- and second-trimester test results are combined (sequential screening). All positive screens are further offered invasive diagnostic tests, such as amniocentesis.

Apart from a contingent sequential screening for the negative first-trimester screen, some centers that could not measure fetal nuchal translucency also preferred second-trimester screening alone. As a result, they did not perform chorionic villus sampling or missed out on first-trimester screening tests. Another vital contribution of the quadruple test is that it includes AFP, a valuable marker for screening for neural tube defects.

Patients in the intermediate screening risk category, i.e., 1:250 up to 1:1000, are offered a more sensitive DNA-based noninvasive prenatal screening (NIPS), which detects the possibility of DS in fetal cells circulating in maternal blood. However, a costly model may prevent the purpose of irrelevant invasive tests [8].

This study protocol was compatible with the Asian population and European studies done in Belgium, the Netherlands, and the UK [9-11].

Invasive/Diagnostic Procedures in the Second Trimester

Amniocentesis for the genetic diagnosis of DS is typically performed at 14-20 weeks; the cytogenetic diagnosis rate is reported to be 99.4% [12]. This procedure is considered safest between 16 and 18 weeks gestation [13].

## Materials and methods

Study design

Retrospective data of 5626 pregnant women were collected as per the inclusion criteria stated in Table 1. The study was conducted between January 2019 and December 2019.

**Table 1:** Inclusion criteria.

S.no	Criteria
1	Singleton pregnancy
2	Unassisted spontaneous pregnancy
3	Gestation age as per fetal measurements of crown rump length or biparietal diameter (BPD) on the date of sample collection, 14–20 weeks and 6 days
4	Asian ethnicity

The study was conducted at the National Reference Laboratory, Apollo Diagnostics. The study was approved by the research council ethics committee of the institution.

Tested biochemical results of samples used in this study were collected from antenatal clinics with written informed consent, which was obtained after counseling for serum maternal screening for fetal aneuploidies, including risks, benefits, and possible outcomes of both false-positive (consideration and implication of invasive diagnostic testing and the possibility that a miscarried fetus may be chromosomally normal) and false-negative screening tests for each patient. 

Samples were transported at 2-8 °C in serum gel tubes to the central reference laboratory for processing within 48 hours of collection.

Analysis and risk calculation for DS

Biochemical analysis was performed within four to six hours of receiving the samples. Delfia immunoflourometry and Victor 2D by Perkin Elmer were used to analyze AFP, free BHcg, and Ue3. A chemiluminescence immunoassay (CLIA) on a Beckman Coulter DXI 800 was used to analyze inhibin-A. After analysis, age risk and final biochemical risk for DS were calculated using Lifecycle version 6.0 (IBM Corp., Armonk, NY). Measurements of biochemical markers were converted into multiples of the median for gestational age and adjusted for maternal weight, smoking, insulin-dependent diabetes, and Asian ethnicity. A screen-positive (SP) result from second-trimester screening was defined as a risk at the end of pregnancy (40 weeks) of less than 1 in 250.

Statistical analysis

Data analysis was done using the statistical software R version 4.1.1 (RStudio, PBC, and Boston, MA) and Microsoft Excel (Microsoft® Corp., Redmond, WA). Categorical variables were represented using numbers and percentages. A Chi-squared test was used to compare proportions across different categories. The proportion of SP cases between the two methods of calculation, i.e., age alone versus final risk calculation with biomarkers, was tested using McNemar’s test to compare nominal levels/binary levels of dependent data. A P-value of ≤0.05 indicates statistical significance.

## Results

The result calculation consisted of the total number of SP and screen-negative (SN) cases according to the age of all pregnancies and the final SP and SN risk after adding biochemical markers.

Data in Table 2 show that of the 5626 pregnancies screened, the majority of women were in the age group 26-30 years (n = 1887). The lowest number was seen in women aged >35 years (n = 302). This may mean: (i) fewer women undergo pregnancy in the advanced age group and (ii) more women opt for invasive diagnostic methods for DS. There is a difference of 42.1% from age-only risk to final SP risk in this population after adding biochemical markers. Age-only risk for DS was seen only in women aged >35 years, as per the software calculation.

**Table 2:** Age groups screened with mean age screen positive risk and mean final SPR. SPR: screen positive risk

Age group (years)	Number of pregnancies screened (n)	Mean final risk SPR (%)	Mean age only SPR (%)
16–20	314	2.6	0
21–25	1573	1.8	0
26–30	1887	2.2	0
31–35	1028	6.2	0
>35	302	43.9	86
Total	5626		

The proportions of SP cases with age risk and final risk were 56.3% and 12.6%, respectively. The computed McNemar’s chi-squared test value was 97.959 (p < 0.001), which indicated a significant reduction in SP cases when biomarkers were added to screen for T21 in women aged >35 years.

Of the total number of women aged >35 years who were screened (n = 302), 170 were screened positive for DS with age risk. After adding biochemical parameters, 78.8% of the total SP tests of age risk screened negative for DS (Table 3).

**Table 3:** Comparison of screen positive risk for DS between age and final risk calculations after addition of biochemical markers in age group above 35 years.

Age risk	Final Risk		Total
	Positive	Negative	
Positive, n (%)	36 (21.2)	134 (78.8)	170
Negative, n (%)	13 (9.8)	119 (90.2)	132
Total	49	253	302

Table 4 shows the screen risk distribution of the SN cases (n = 134) of DS. From the age-risk-positive group of age >35 years (n = 302), borderline cases were segregated, with risk ratios of up to 1:500 and 1:1000 (26.1% and 23.1%, respectively). This indicates that of the total SN cases, including borderline cases up to 1:1000, 49.2% can still be offered NIPS. At least 50% of the SN cases could avoid an invasive test.

**Table 4:** Final risk screen negative n=134 cases from total age risk positive cases: risk ratio distribution.

Final risk 1:XXX	n	%
>250–500	35	26.1
>500–1000	31	23.1
>1000	68	50.8
Total	134	

## Discussion

Three types of genetic variations result in DS, of which meiotic nondisjunction is found in approximately 95% of all cases. The recurrence risk of this predisposition is linked with advanced maternal age (Table 5) [14].

**Table 5:** Genetic basis of Down syndrome.

Genetic mechanism	Abnormality	Recurrence in population
Meiotic nondisjunction (92 –95%)	Extra copy of chromosome 21 present in all cells	Based on maternal age
Mosaicism trisomy 21 (2–4%)	Extra copy of chromosome 21 present in some cells	Similar to the healthy population
Robertsonian/unbalanced translocation (3–4%)	All or part of chromosome 21 attached to another chromosome	High if balanced translocation is present in one parent

An estimated 75% of affected fetuses are born to mothers aged <35 years at delivery, although the risk of DS increases with maternal age. It is imperative to note that an increasing number of women are opting for planned pregnancy after 35 years of age.

Pregnant women of all ages are offered screening and invasive diagnostic testing for chromosomal abnormalities before 20 weeks of gestation. Since advanced maternal age itself is a risk factor for DS, few countries have made it mandatory for these women to undergo invasive testing such as amniocentesis, which carries a risk of fetal loss of about 1-2%. Vaginal spotting, amniotic fluid leakage, fetal needle injury, and fetal loss may be other complications of invasive techniques [15].

In a study conducted on 35,236 women enrolled for second-trimester screening, 87 cases with DS were identified, of which only 64 (21.6%) fetuses were detected in pregnancies with maternal age ≥35 years [7]

A genetic counseling study comprising 3500 subjects in a tertiary genetic center revealed that only 28.7% of DS cases were identified in prenatal diagnosis in India. For cases with advanced maternal age (35.7%), indications for amniotic fluid studies were highest, whereas other indications included an abnormal second-trimester test (21.3%), a previous child with T21 (21.3%), and abnormalities seen on ultrasound (11.1%) [16].

Furthermore, another study showed that maternal age was the determinant of the risk of DS, and women aged >35 years were offered genetic counseling and amniocentesis. Nevertheless, only 20% of infants with DS were born to women aged >35 years [15-17].

This study has indicated that only 21.2% had a final SP risk of DS from the total number of high maternal age risk, and 49.2% of the SN cases had an intermediate risk of up to 1/1000. A screening test is important for examining the high-risk population, which should be further managed through invasive procedures.

Limitations

Although the advantages of antenatal screening include increasing the odds of identifying an abnormal fetus and reducing the number of invasive diagnostic tests and procedure-related losses of normal fetuses, the disadvantage of screening is that not all aneuploid fetuses can be identified with screening. The data of SP or SN were not followed-up for outcomes

## Conclusions

This study has highlighted that women aged >35 years showed an SP risk of DS if age alone is considered a risk factor for DS. However, with the addition of biochemical parameters, a significant proportion of this age group screened negative. Thus, subjecting pregnancies, particularly precious or late pregnancies that have a probability of a normal fetus, directly to invasive procedures that have a 1-2% risk of fetal loss should be reconsidered.

Therefore, advanced maternal age should not be the only criterion for offering an invasive diagnostic test. Women of all age groups should be screened using biochemical serum tests and or NIPS regardless of age, including those aged >35 years, to rule out DS before considering an invasive diagnostic test.
